# Harnessing Polyaminal Porous Networks for Sustainable Environmental Applications Using Ultrafine Silver Nanoparticles

**DOI:** 10.3390/polym17182443

**Published:** 2025-09-09

**Authors:** Bedour Almalki, Maymounah A. Alrayyani, Effat A. Bahaidarah, Maha M. Alotaibi, Shaista Taimur, Dalal Alezi, Fatmah M. Alshareef, Nazeeha S. Alkayal

**Affiliations:** 1 Chemistry Department, Faculty of Science, King Abdulaziz University, P.O. Box 80203, Jeddah 21589, Saudi Arabia; bturaykhimalmalki@stu.kau.edu.sa (B.A.); malrayyani@kau.edu.sa (M.A.A.); ebahaidarah@kau.edu.sa (E.A.B.); mmsalotaibi@kau.edu.sa (M.M.A.); dalezi@kau.edu.sa (D.A.); fmalshareef@kau.edu.sa (F.M.A.); 2 Department of Chemistry, Pakistan Institute of Engineering and Applied Science, PO Nilore, Islamabad 44000, Pakistan; shaista.taimur@pieas.edu.pk

**Keywords:** melamine-based porous polyaminals, silver nanoparticles (Ag NPs), reverse double-solvent method, CO_2_ capture, heavy metal removal

## Abstract

Environmental contamination is a critical global concern, primarily due to detrimental greenhouse gas (GHG) emissions, especially carbon dioxide (CO_2_), which significantly contribute to climate change. Moreover, the presence of harmful heavy metals like Ni, Cd, Cu, Hg, and Pb in soil and water ecosystems has led to poor water quality. Noble metal nanoparticles (MNPs), for instance, Pd, Ag, Pt, and Au, have emerged as promising solutions for addressing environmental pollution. However, the practical utilization of MNPs faces challenges as they tend to aggregate and lose stability. To overcome this issue, the reverse double-solvent method (RDSM) was utilized to synthesis melamine-based porous polyaminals (POPs) as a supportive material for the in situ growing of silver nanoparticles (Ag NPs). The porous structure of melamine-based porous polyaminals, featuring aminal-linked (-HN-C-NH-) and triazine groups, provides excellent binding sites for capturing Ag^+^ ions, thereby improving the dispersion and stability of the nanoparticles. The resulting material exhibited ultrafine particle sizes for Ag NPs, and the incorporation of Ag NPs within the porous polyaminals demonstrated a high surface area (~279 m^2^/g) and total pore volume (1.21 cm^3^/g), encompassing micropores and mesopores. Additionally, the Ag NPs@POPs showcased significant capacity for CO_2_ capture (2.99 mmol/g at 273 K and 1 bar) and effectively removed Cu (II), with a remarkable removal efficiency of 99.04%. The nitrogen-rich porous polyaminals offer promising prospects for immobilizing and encapsulating Ag nanoparticles, making them outstanding adsorbents for selectively capturing carbon dioxide and removing metal ions. Pursuing this approach holds immense potential for various environmental applications.

## 1. Introduction

Nowadays, the world is facing a vital environmental challenge, and one of the prominent issues is the phenomenon of global warming and climate change. These problems are the result of growth in industries and human activities [1,2]. To meet the rising demands for energy consumption, transportation, and water treatment, there is a strong reliance on fossil fuels as a primary source of energy. Fossil fuels such as coal, oil, and gas are burned, releasing significant amounts of greenhouse gases (GHGs) into the atmosphere [3,4,5]. Among these gases, carbon dioxide (CO_2_) is particularly responsible for the rise in average global temperatures [6,7,8,9]. Excessive CO_2_ emissions, accounting for about 80% of all greenhouse gas emissions, have been usually documented as the main reason for global warming [10,11]. The rapid increase in CO_2_ concentration globally can have numerous detrimental effects, including ocean acidification, polar ice melting, rising sea levels, droughts, and hurricanes. These effects pose threats to human health and the global economy [3,12].

Water pollution is another pressing environmental problem that presents significant challenges. Water is a fundamental resource necessary for sustaining life on earth, and ensuring access to clean water is crucial for both humans and the ecosystem. Unfortunately, the quality of water has been adversely impacted by the continuous pollution caused by a combination of human and natural factors. Numerous human activities, such as urbanization, population growth, industrial production, climate change, and other contributing factors, directly influence the quality of water [13,14,15].

One of the major threats is water contamination by heavy metals, including copper (Cu), zinc (Zn), cadmium (Cd), cobalt (Co), chromium (Cr), lead (Pb), nickel (Ni), and manganese (Mn). These heavy metals are prevalent in the environment and pose risks to both the ecological environment and human health, even at low concentrations [16,17]. Heavy metals enter the environment as stable and relatively non-degradable contaminants, leading to their accumulation in living organisms through the process of bioaccumulation. Over time, the levels of these metals increase in organisms and can accumulate throughout the food chain. This accumulation results in toxicity and various health hazards, which can be particularly dangerous to humans [18,19]. Copper contamination, for example, can lead to insomnia, Wilson’s disease, and liver damage. Cadmium, commonly used in industries like plastics and batteries, can cause kidney damage, carcinogenic effects, and renal disorders, and contribute to emphysema. Lead pollution, often associated with battery factories, can result in brain damage, anemia, anorexia, vomiting, and diseases affecting the circulatory and nervous systems. Exposure to nickel can lead to dermatitis, nausea, chronic asthma, coughing, and lung cancer [13,20,21].

Science and technology play a crucial role in developing innovative approaches, tools, and techniques to address environmental problems related to air, water, and wastewater. In the realm of air and water pollution, nanotechnology is considered to be a very promising method for pollutant removal and detection. Metal nanoparticles (MNPs), particularly noble metals like Ag, Au, and Pd, have demonstrated remarkable potential in remediating various environmental pollutants. This is due to the unique chemical and physical properties exhibited by nanomaterials, such as their large surface area, composition, electrical properties, magnetic properties, mechanical properties, and optical properties [22,23,24,25]. However, a significant challenge in working with MNPs lies in achieving controllable synthesis, since these nanoparticles are thermodynamically unstable and naturally prone to aggregation, especially at nano sizes in the ultrafine range, due to their high surface energy. This aggregation issue is one of the major hurdles faced in the field of MNPs [23,26,27,28]. 

Recent studies have focused on utilizing porous materials as supports for metal nanoparticles (MNPs) to either encapsulate or disperse them [28,29,30]. These porous materials, including metal–organic frameworks (MOFs), graphene oxide (GO), zeolites, mesoporous silica, graphitic carbon nitride (g-C_3_N_4_), and porous organic polymers (POPs), play crucial roles in preventing the agglomeration of MNPs and enhancing their dispersion [22,27,28,31,32]. Not only do these porous materials stabilize the MNPs through coordination, confinement effects, and electrostatic interactions, but they also impart unique physicochemical properties to the MNPs. Additionally, they effectively improve the stability and activities of the metal nanoparticles [29,33]. 

Porous organic polymers (POPs), including melamine-based polyaminals, have attracted important consideration and research interest as a result of their wide range of potential applications such as separation, water treatment, gas storage, photoelectric conversion, chemical and bio-sensing, heterogeneous catalysis, and energy storage and conversion. POPs have emerged as highly promising adsorbents for environmental pollutant removal due to their numerous advantages and beneficial characteristics [34,35]. These include the following: (1) They have permanent porosity, facilitating rapid mass transfer, and high specific surface areas, and their abundant binding sites enhances their adsorption capacity [34,36]. (2) They have exceptional chemical stability, enabling them to withstand harsh environmental conditions [37]. (3) Their structural flexibility allows for the easy design and synthesis of various POPs with rational functional groups through the employment of various building blocks and advancements in reticular chemistry [38]. (4) They have a low density, resulting from the predominant presence of lightweight elements (such as N, C, H, O, B, etc.), leading to exceptional gravimetric performance and high adsorption capacity [35,39]. (5) They have diverse organic synthesis strategies and the capability to be synthesized from a varied range of monomers with strong covalent bonds linking them together [40,41]. 

Melamine-based porous polyaminals have emerged as a promising class of POPs due to their simplified synthesis method, customizable structure, high surface area, nitrogen-rich composition, and thermal stability [42,43,44]. Melamine, being readily available and cost-effective, serves as an excellent building block for POP development with its multiple nitrogen sites, amino groups, and stable triazine ring. The inherent basic nature of melamine, in conjunction with aldehyde derivatives, enhances its reactivity in forming aminal linkages (-HN-C-NH-) [45,46,47,48,49]. Fortunately, it has been noticed that POPs have great potential as a perfect support for metal nanoparticles [50]. The combination of heteroatom scaffolds, such as nitrogen, help guide the nucleation, capture of metal ions, and growth of metal nanoparticles, ultimately preventing their aggregation [30,31,33,51]. Overall, these features make porous organic polymers, especially melamine-based porous polyaminals, highly suitable for stabilizing metal nanoparticles and enhancing their interactions with the POP support [26,52,53]. The incorporation of metal precursors into the pores of porous organic polymers (POPs), such as melamine-based porous polyaminals, can be achieved through a widely used and straightforward method known as the reverse double-solvent method [54,55]. This method involves two main steps. Initially, a hydrophilic solvent containing metal precursors is mixed with a large amount of a hydrophobic solvent to surround the POPs. The volume of the hydrophilic solvent used is smaller than the pore volume of the POPs. Subsequently, the metal precursors undergo a reduction process inside the pores of the POPs using reducing agents like H_2_ or NaBH_4_. This reduction process leads to the formation of metal nanoparticles (MNPs) within the cavities of the POPs [23,56,57].

Based on the above considerations, this paper reports the successful synthesis of novel melamine-based porous polyaminals doped with silver nanoparticles (Ag NPs@Bipy-PAN) for the stable immobilization of silver NPs. The melamine-based porous polyaminals exhibited a high surface area and had a porous structure consisting of micropores and mesopores. These polymers contained nitrogen-rich cages with triazine and bipyridine groups, which effectively regulated the growing of ultrafine silver nanoparticles and encapsulated them within the pores. The resulting material, Ag NPs@Bipy-PAN, comprised highly dispersed silver nanoparticles with an average particle size of 9.2 nm, which were securely immobilized within the pores of the Bipy-PAN support. This nanoadsorbent displayed remarkable adsorption capacity for CO_2_ and demonstrated efficient removal of Cu (II) ions.

## 2. Materials and Methods

### 2.1. Materials

The chemicals used in the experiment were supplied by commercial sources and used without further purification. Tetrahydrofuran (THF ≥ 99.5%), acetone (99.5%), dichloromethane (CH_2_Cl_2_ ≥ 99.9%), and Pd(NO_3_)_2_ (99%) were purchased from Fisher Scientific in Chicago, USA. NaOH (98%), NaBH_4_ (99%), and (CH_3_.COO)_2_ Cd.2H_2_O (99%) were supplied by BDH Chemicals in London, UK. [2,2′-Bipyridine]-5,5′-dicarbaldehyde was purchased from Shanghai Sunchem Inc. in Shanghai, China. Melamine (97.5%), silver acetate (CH_3_COOAg 98.5%), and dimethyl sulfoxide (DMSO 99%) were supplied by BDH Laboratory Reagents in London, UK. NiSO_4_.6H_2_O (98%) and (CH_3_COO)_2_ Cu.H_2_O (≥99.8%) were obtained from BDH Laboratory Supplies in London, UK. Ward’s Natural Science in Rochester, NY, USA, supplied Ba(NO_3_)_2_ (99%), while LOBA Chemie in Mumbai, India, supplied HCl (35%).

### 2.2. Synthesis of the Ag NPs@Bipy-PAN

In order to synthesize silver nanoparticles encapsulated in porous organic polymer, first, the bipyridine-based polyaminal-linked porous polymer (Bipy-PAN) was synthesized as reported previously [58]. A total of 30 mL of DMSO was used to dissolve 0.5 g of melamine (3.96 mmol) and 0.7 g of [2,2’-Bipyridine]-5,5’-dicarbaldehyde (5.94 mmol). The reaction mixture was kept under argon and heated at 175 °C for 72 h. The precipitate was rinsed three times with acetone, three times with dichloromethane, and three times with dimethylformamide. The cured product was then vacuum-dried for two hours at 70 °C, yielding a 72% yield.

**Scheme 1:**
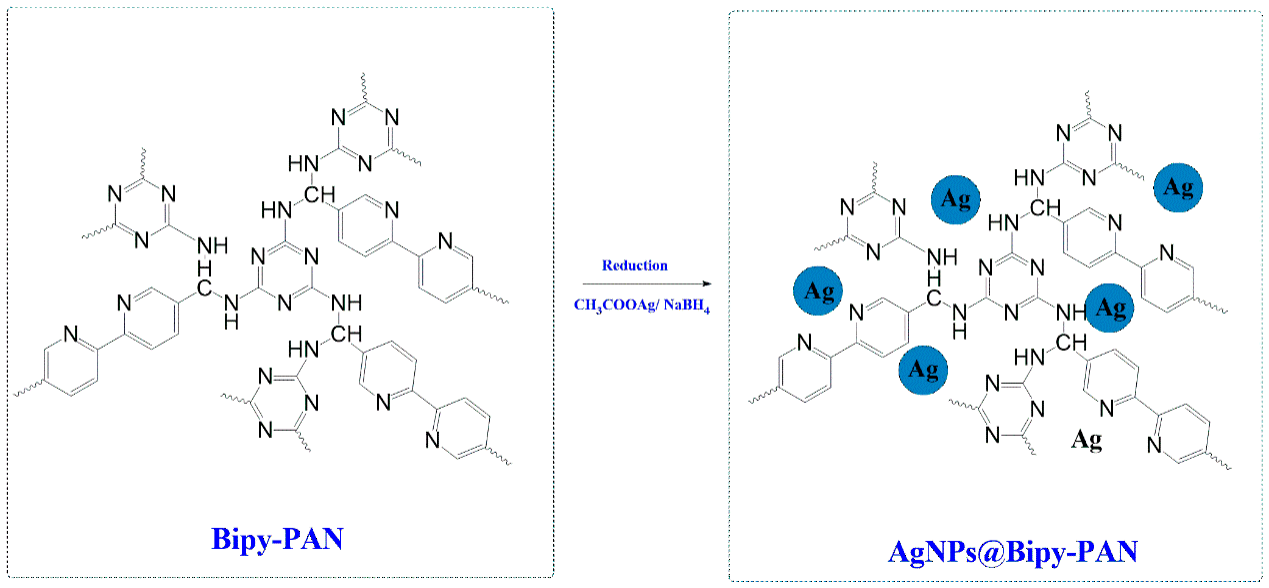
Preparation of Ag NPs@Bipy-PAN.

Ag NPs were encapsulated by Bipy-PAN as described previously [59]. A total of 170 mg of Bipy-PAN was dispersed in 40 mL of deionized water as a hydrophilic solvent, and the solution was sonicated for 1 h until achieving homogeneity. Following 30 min of stirring, a solution of CH_3_COOAg (0.02 mmol) dissolved in CH_2_Cl_2_ (0.04 mL) as the hydrophobic solvent was slowly added dropwise over 10 min with continuous vigorous stirring. The mixture was continuously stirred for five hours as a result. The prepared mixture was then reduced by the addition of 1 mL of a high-concentration aqueous solution of NaBH_4_ (2.7 M). The mixture was eventually filtered, washed with deionized water, and dried at 70 °C for two hours to produce the solid sample, as shown in Scheme 1.

### 2.3. Characterization Methods

Powder X-ray diffraction (PXRD) analysis was performed utilizing a Bruker D8 Advance instrument with Cu K α radiation (λ = 1.5418 Å) to ascertain the structure of the solid phase and the crystallinity of Ag species. The measurements were performed at room temperature, collecting patterns in the 2θ range of 10° to 80°. Thermogravimetric analysis (TGA, TG-DTA6300) was employed to assess the thermal stability of the material under a N_2_ atmosphere. The analysis was conducted with a heating rate of 10 °C/min in the temperature range of 25–500 °C. The microstructure, morphology, and the distribution and agglomeration state of the metal Ag NPs in the prepared Ag NPs@Bipy-PAN sample were examined using scanning electron microscopy (SEM), transmission electron microscopy (TEM), and energy dispersive X-ray spectroscopy (EDX). The SEM and EDX imaging were carried out using the FEI TENEO vs. microscope equipped with an EDAX detector. The Brunauer–Emmett–Teller (BET) specific surface area and the Barrett–Joyner–Halenda (BJH) pore-size distribution of the samples were determined by N_2_ adsorption–desorption measurements at 77 K using a Micromeritics 3 Flex 3500 analyzer. Before measurement, the samples were subjected to drying under vacuum at 120 °C for 12 hours. The CO_2_ capacity was evaluated at 273 K up to 1 bar. The metal contents in the solution were determined using inductively coupled plasma atomic emission spectroscopy (ICP-AES) with a Perkin-Elmer Optima 7000 DV instrument. Finally, field emission scanning electron microscopy (SEM, JSM-IT500HR) and energy dispersive X-ray spectroscopy mapping (EDX, STD-PC80) were utilized to investigate the structure, morphology, and elemental composition of Ag NPs@Bipy-PAN@Cu (II).

### 2.4. Adsorption of Heavy Metal Ions

Appropriate amounts of metal salts were used to prepare a stock solution of 20 mg/L of each metal in distilled water, including Ba (II), Ni (II), Pb (II), Cu (II), and Cd (II). A total of 100 mL of the solution was added to a bottle containing 20 mg of Ag NPs@Bipy-PAN, the pH was adjusted to be 6, and the mixture was shaken in a water bath shaker at 200 rpm for an hour at room temperature. After filtering the mixture, the filtrate was gathered, and the ion concentration was determined by ICP-AES. The adsorption capacity (*q_e_*) and the removal efficiency (R%) were calculated using the following equations:

(1) $$q_{e\;}=\frac{C_{e}-C_{f}}{m}\times V$$

(2) $$R\%=\frac{C_{e}-C_{f}}{C_{e}}\times 100$$

where $C_{e}\;$ and $C_{f}$ are the initial and the final concentrations of metal (mg/L), *V* is the volume of the solution (L), and m is the mass of the adsorbent (g).

#### 2.4.1. Effect of pH on Metal Ion Adsorption

A total of 20 mg of Ag NPs@Bipy-PAN was added to each of the five 100 mL of Cu (II) solutions (20 mg/L). The pHs of the solutions were adjusted to 4, 5, 6, 7, and 8 using 0.01 M HCl and 0.01 M NaOH. The mixtures were shaken in a water bath shaker at 200 rpm for an hour at room temperature. The filtrates were collected and tested by ICP-AES.

#### 2.4.2. Effect of Polymer Dosage on Metal Ion Adsorption

A 100 mL solution of 20 mg/L Cu (II) was mixed with varying Ag NPs@Bipy-PAN masses (10, 20, 30, 40, and 50 mg), and the mixtures were shaken at 200 rpm for an hour at the optimum pH and room temperature. The filtrates were collected and tested by ICP-AES.

#### 2.4.3. Effects of Initial Concentration on Metal Adsorption

A total of 30 mg of Ag NPs@Bipy-PAN was added to 100 mL of Cu (II) solution in different concentrations (10, 20, 50, 150, and 200 ppm) of solution. At optimum pH, the mixtures were shaken at 200 rpm for an hour at room temperature. The filtrates were collected and tested by ICP-AES.

#### 2.4.4. Effects of Contact Time on Metal Ion Adsorption

At the optimum pH, a mixture of 20 mg/L Cu (II) solution and 30 mg of Ag NPs@Bipy-PAN was shaken at 200 rpm at room temperature. The samples were collected and analyzed by ICP-AES at regular intervals from 20 min to 120 min. 

#### 2.4.5. Desorption and Regeneration Study

The desorption studies were carried out using 50 mL of (0.5 M) HCl. After the adsorption process of 20 mg/L of Cu (II) in 30 mg on Ag NPs@Bipy-PAN at the optimum pH, the mixture was filtered, and the filtrate was analyzed. Then, the nanoadsorbent was washed with 50 mL HCl and deionized water, then reused. The adsorption–desorption cycle was performed four times for the measurement.

## 3. Results and Discussion

The Bipy-PAN polymer effectively stabilizes metal ions through the coordination at the nitrogen atoms of its bipyridine, triazine, and aminal linkage units. This highlights its strong potential as a support for immobilizing metal nanoparticles. PXRD has verified the successful encapsulation of Ag NPs in the Bipy-PAN polymer. Ag NPs incorporated into Bipy-PAN exhibited a broad diffraction hump at 2θ = (21°), confirming the amorphous character of the Bipy-PAN polymer. Moreover, a sharp diffraction peak at 2θ = (38.2°) was detected, which corresponds to the (111) planes of the face-centered cubic lattice (FCC) of Ag nanoparticles (JCPDS No. 04-0783) [58,59]. These results demonstrate that the Ag nanoparticles were uniformly dispersed and confined in the Bipy-PAN matrix without inducing major changes to its inherent structure [22,56,59].

### 3.1. Morphological Analysis of Ag NPs Incorporated into Bipy-PAN

**Figure 1:**
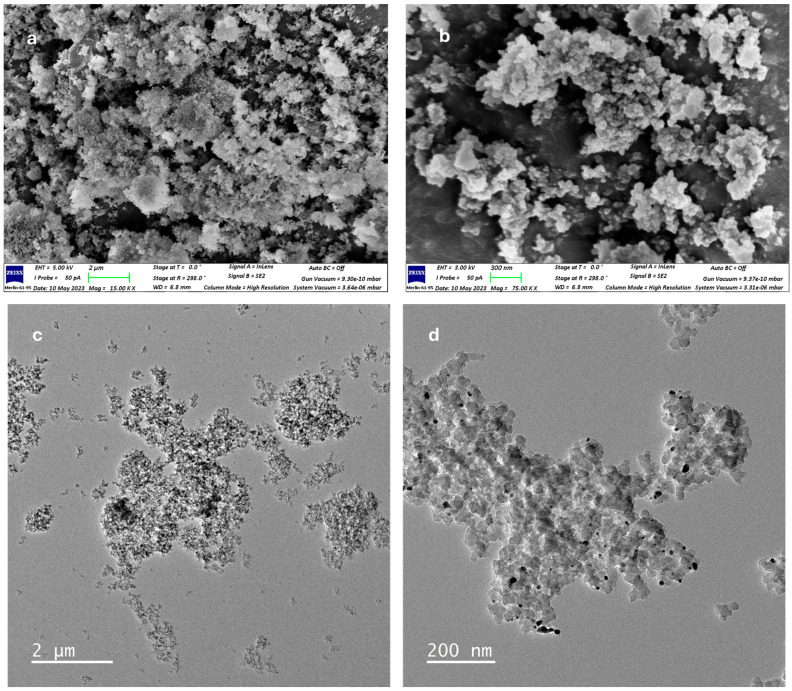

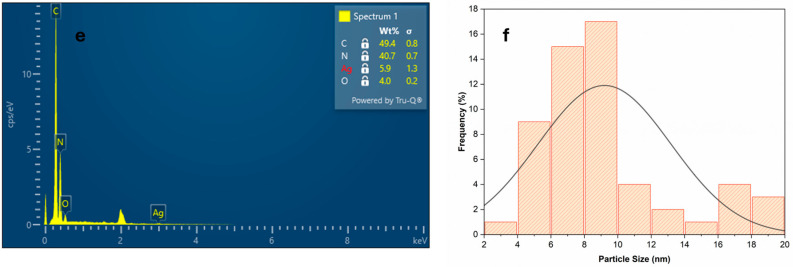
(**a**,**b**) SEM images of Bipy-PAN-supported Ag NPs with different magnifications, (**c**,**d**) TEM images of Bipy-PAN-supported Ag NPs with different magnifications. (**e**) EDX analysis of Bipy-PAN-supported Ag NPs and (**f**) size distribution histogram.

SEM and TEM analysis thoroughly investigated the material’s morphology, structural features, and particle size distribution [59]. The compact and irregular shape of Ag NPs incorporated into Bipy-PAN was observed in the SEM image (Figure 1a,b). Interestingly, the Bipy-PAN capsulation with Ag NPs maintained its original morphology even after the incorporation of nanoparticles. TEM analysis (Figure 1c,d) confirmed a spherical Bipy-PAN structure, with the Ag nanoparticles visible as black dots uniformly distributed across its surface. The average Ag NP diameter was measured at 9.2 nm, a size attributed to the coordination between the Ag nanoparticles and the nitrogen active sites within the Bipy-PAN polymer (Figure 1e,f). Furthermore, the EDX analysis proved the presence of uniformly distributed C, N, and Ag on the sample surface (Figure 1e,f). These findings confirm the effective anchoring of Ag nanoparticles onto the Bipy-PAN polymer matrix, without inducing notable alterations in its morphology [59].

### 3.2. BET and BJH Analysis

**Figure 2:**
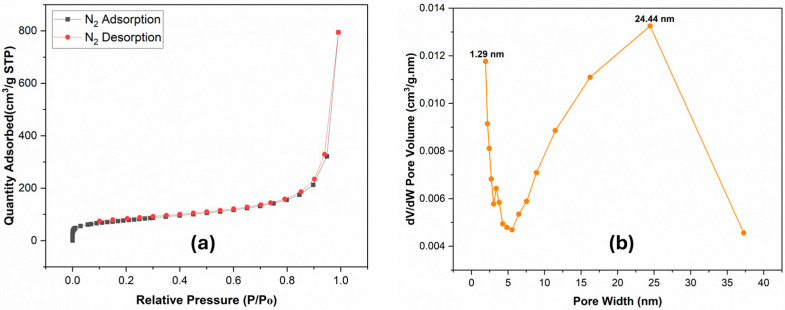

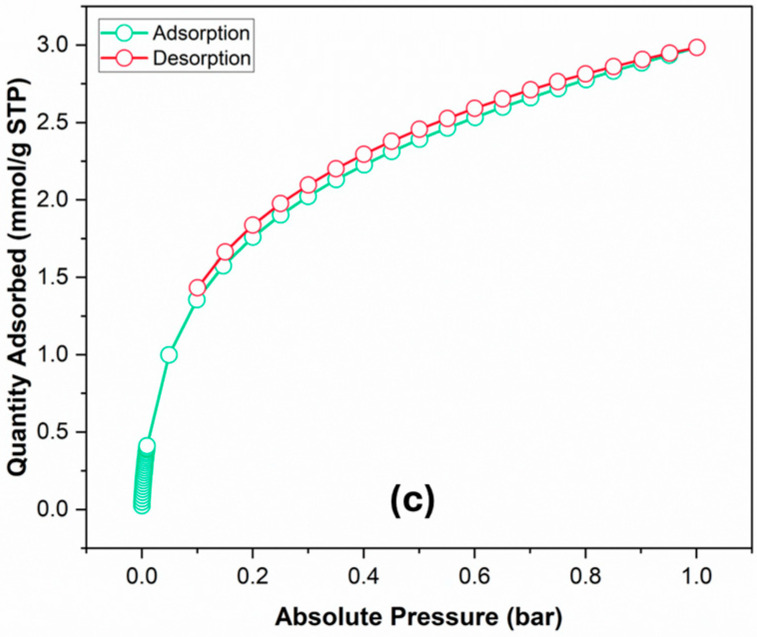
(**a**) N_2_ adsorption–desorption isotherms, and (**b**) porosity distribution via the original density functional theory of Ag NPs@Bipy-PAN (**c**) CO_2_ uptake isotherms of Ag NPs@Bipy-PAN.

The nitrogen (N_2_) adsorption at 77 K experiment was used to investigate the porosity of the Bipy-PAN-supported Ag NPs. As shown in Figure 2a, Ag NPs@Bipy-PAN displays a representative type IV adsorption isotherm [60]. The N_2_ adsorption–desorption isotherms of the Ag NPs@Bipy-PAN manifest a rapid growth of N_2_ uptake in a low relative pressure range P/P_0_ < 0.002, demonstrating the existence of a 49.1522 m^2^/g micropore area. The presence of mesopores is evidenced by the hysteresis loop that accompanies the desorption curve. The total pore volumes were calculated at P/P_0_ = 0.99 and found to be 1.21 cm^3^/g. Ag NPs@Bipy-PAN’s BET specific surface area was calculated to be ~279 m^2^/g, while Bipy-PAN specific surface areas were ~161 m^2^/g [58]. This indicates that the (BET) specific surface areas were improved because of ultrafine Ag NP loading inside the pores of Bipy-PAN [27,61]. The ultrafine Ag NPs possess a greater surface-to-volume ratio, which results in Ag NPs@Bipy-PAN having an increased surface area due to a higher number of active surface sites. Furthermore, the BJH model is applied to estimate how pore sizes are distributed in Ag NPs@Bipy-PAN. As shown in Figure 2b, the BJH model of Ag NPs@Bipy-PAN indicates a predominate presence of micropores and mesopores, with pore sizes centered at approximately 1.29 nm and 24.44 nm, respectively.

### 3.3. CO_2_ Adsorption

The CO_2_ adsorption isotherms of Ag NPs@Bipy-PAN were recorded at 273 K with a pressure of 1 bar (Figure 2). It is observed that the CO_2_ uptakes ranged between 0.02 and 2.99 mmol/g at 273 K, suggesting an exothermic physical adsorption process. The maximum CO_2_ adsorption capacities for Ag NPs@Bipy-PAN and Bipy-PAN at 1 bar and 273 K were 2.99 and 1.02 mmol/g, respectively [58]. It was noticed that the CO_2_ adsorption by Ag NPs@Bipy-PAN was superior. This can be attributed to the increased pore volume resulting from the introduction of Ag nanoparticles, which enhanced the porosity of Bipy-PAN. Consequently, the pore width improved, leading to the formation of a microporous structure. The higher proportion of micropores in the network proved advantageous for the adsorption of small molecule gases such as CO_2_. These results underscore the significant role of micropores in the network, influencing the CO_2_ adsorption capacity [28,62]. One should note that the CO_2_ adsorption of Ag NPs@Bipy-PAN surpassed that of several other melamine-based porous polyaminals reported in the literature (Table 1).

### 3.4. Metal Adsorption

The adsorption capacity of the polymers Bipy-PAN and Ag NPs@Bipy-PAN toward metal cations was studied, and the results are shown in Table 2. The Ag NPs@Bipy-PAN polymer shows better adsorption behavior than the Bipy-PAN polymer. This indicates that successfully loading Ag NPs into the Bipy-PAN polymer material provides a high specific surface area, leading to an increase in the adsorption rate caused by effective Ag atom utilization [65]. Furthermore, during the adsorption study, it was found that the removal efficiency of Cu (II) was greater compared to the other cations. Therefore, the Cu (II) cation was chosen to undergo further adsorption studies (Table 2).

**Figure 3:**
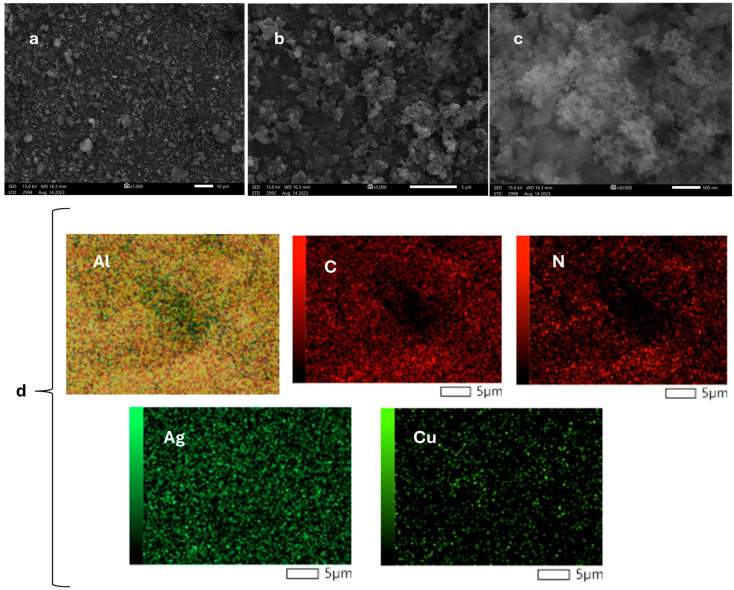
(**a**–**c**) SEM images of Ag NPs@Bipy-PAN with various magnifications and (**d**) EDX mapping of Ag NPs@Bipy-PAN.

Moreover, Cu (II) adsorption on Ag NPs@Bipy-PAN was confirmed through SEM and EDX analysis. SEM was utilized to visualize the morphology of Ag NPs@Bipy-PAN after adsorbing Cu (II). The captured micrographs revealed a layered and porous structure at different magnifications. Notably, SEM demonstrated that the morphology of Ag NPs@Bipy-PAN remained intact even after copper ion adsorption (Figure 3a–c). Furthermore, EDX elemental mapping confirmed the presence of C, N, and Ag elements, as well as the effective dispersion of Cu (II) within Ag NPs@Bipy-PAN (Figure 3d). The findings from the study indicated that Ag NPs@Bipy-PAN exhibited a remarkable adsorption capacity for Cu (II).

#### 3.4.1. pH Influence

**Figure 4:**
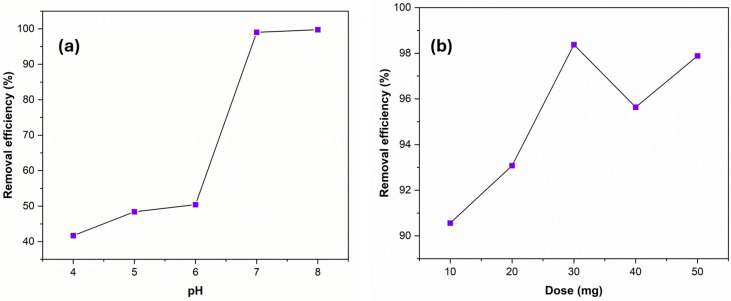
The effect of pH (**a**) and adsorbent dose (**b**) on the removal efficiency of 20 mg/L Cu (II) solution.

Studying the effect of pH of the aqueous solution is an important investigation that significantly impacts the adsorption process of an adsorbent. Moreover, the pH of the solution may affect the degree of ionization, speciation of the adsorbent, the surface charge of the adsorbents, and metallic ion hydro-complexes. The pH value of the mixture solution was adjusted from 4 to 8 by using HCl or NaOH. Figure 4a shows the strong effect of pH solution on the removal efficiency of Cu (II) due to the competition between H^+^ and OH^−^ ions and Cu (II) ions on the active sites of the sorbent polymer in acidic or basic solutions. In the acidic media, the active nitrogen sites were deactivated with protons; therefore, the removal efficiency of Cu (II) is ineffective at an acidic pH value. Additionally, at a pH higher than 7, the precipitation of Cu (II) ions as Cu(OH)_2_ can be motivated, which is not favorable. As a result, pH 7 was applied in further adsorption experiments. 

#### 3.4.2. Effect of Polymer Dose

The dose effects in this context pertain to how the quantity or dosage of the adsorbent (Ag NPs@Bipy-PAN) affects the adsorption of Cu (II) ions. It is important to investigate the dose effects to determine the optimal amount of adsorbent needed to achieve the desired level of adsorption efficiency. As depicted in Figure 4b, the efficiency of Cu (II) removal increased as the dosage of the adsorbent increased in a constant concentration of Cu (II) solution. This improvement can be attributed to the availability of a larger surface area when a larger amount of the adsorbent is used, leading to more active sites for the adsorption process. However, there is a saturation point at which the polymer can no longer adsorb additional Cu (II) ions, regardless of the quantity of adsorbent applied. Notably, a dosage of 30 mg of the adsorbent yielded favorable outcomes in the adsorption experiment. Table 3 provides a comparison of the adsorption capacity of Ag NPs@Bipy-PAN with other melamine-based porous polyaminal adsorbents. The modification of PAN with Ag NPs enhances its adsorption performance toward Cu (II) ions by introducing additional active sites and facilitating stronger interactions. The bipyridine groups in the PAN matrix coordinate with Ag NPs, creating a surface rich in binding sites. Cu (II) ions can interact with these sites via electrostatic attraction, coordination with nitrogen atoms, and possible π–metal interactions with the bipyridine ligands. This synergistic effect between PAN, bipyridine ligands, and Ag NPs leads to improved Cu (II) uptake compared to unmodified PAN.

#### 3.4.3. Adsorption Isotherm Study

**Figure 5:**
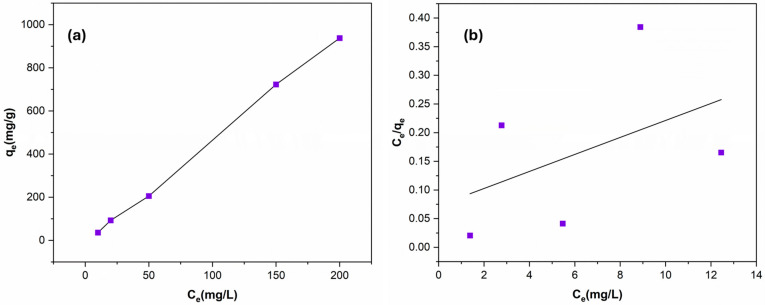
The influence of Cu (II) ion initial concentration on the adsorption capacity (**a**) and the plot of the Freundlich isotherm model (**b**) at pH = 7 and a 30 mg adsorbent dose.

Figure 5a illustrates how the adsorption process by Ag NPs@Bipy-PAN is affected by the initial concentration of Cu (II) ions in the range of 10–200 mg/L at room temperature. As anticipated, the capacity of the adsorption increases with a rising solution concentration, until the polymer approaches saturation. To determine the surface characteristics and adsorbent/adsorbate affinity, the adsorption data were investigated using both the Langmuir and Freundlich models. The Langmuir isotherm model (Equation (3)) assumes that adsorption takes place homogeneously across independent active sites on the adsorbent surface, forming monolayer coverage [67]:

(3) $$\frac{C_{e}}{q_{e}}=\frac{C_{e}}{q_{m}}+\frac{1}{bq_{m}}$$

where $C_{e}$ is the concentration at equilibrium of Cu (II) in the solution represented in mg/L, $q_{e}\;$ is the Cu (II) uptake by the adsorbent at equilibrium concentration (mg/g), $q_{m\;}$ is the uptake capacity of the adsorbent expressed in (mg/g), and *b* represents the Langmuir constant (L/mg). On the other hand, the Freundlich model (Equation (4)) describes the adsorption of Cu (II) over a reversible heterogeneous surface of the adsorbent, where the process is not limited to monolayer adsorption [67]:

(4) $$logq_{e}=logk_{f}+\frac{1}{n}logC_{e}$$

where the constants $k_{f}$ and 1/*n* represent the adsorption capacity and adsorption intensity, respectively. Table 4 summarizes the calculated parameters for both models and according to the correlation coefficients (*R*^2^), the Freundlich sorption model shows a better agreement with the isotherm data (Figure 5b). The *R*^2^ value of 0.936 indicates a strong correlation, suggesting that the inner surface of Ag NPs@Bipy-PAN is more heterogeneous and capable of adsorbing Cu (II) cations in multiple layers [68]. Furthermore, the value of (*n*), which corresponds to the adsorption strength and reflects the degree from linearity for Ag NPs@Bipy-PAN, is calculated to be 3.042, which is exceeding 1. This value indicates that the adsorption processes involve physisorption [69,70].

#### 3.4.4. Kinetics Study

**Figure 6:**
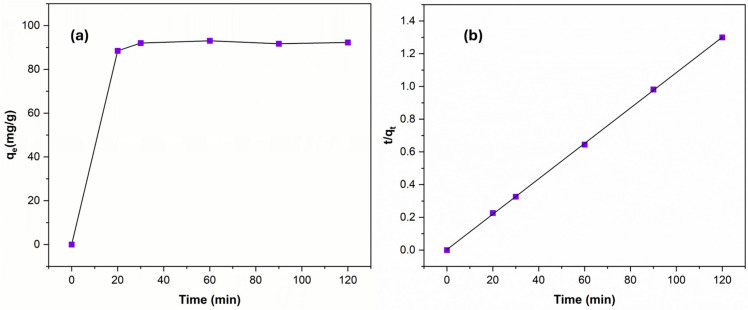
The effect of adsorption time (**a**) and the plot of the pseudo-second-order model (**b**) of 30 mg Ag NPs@Bipy-PAN in 20 mg/ of Cu (II) at pH = 7.

The kinetics behavior of the adsorbent was studied using 20 ppm of Cu (II) and 30 mg of Ag NPs@Bipy-PAN at the optimum pH by collecting samples at periods of 20, 30, 60, 90, and 120 min. As it appears in Figure 6a, the adsorption equilibrium has been achieved in the first 20 min, which indicates the rapid Cu (II) adsorption efficiency by Ag NPs@Bipy-PAN. The observed experimental values were fitted to kinetic models based on the pseudo-first order (PFO, (Equation (5)) and pseudo-second order (PSO, Equation (6)) models, and the corresponding parameters are summarized in Table 5 [71].

(5) $$log\;\left(q_{e}-q_{t}\right)=logq_{e}-\frac{k_{1}t}{2.303}$$

(6) $$\frac{t}{q_{t}}=\frac{t}{q_{e}}+\frac{1}{k_{2}q_{e}^{2}}$$

where $q_{e\;}$ and $q_{t}$ are the adsorption capacity (mg/g) at equilibrium and time *t*, respectively, and $k_{1}$ and $k_{2}$ are the first-order adsorption rate constant (${min}^{-1}$) and second-order adsorption rate constant (g/mg·min), respectively. It was found that the PSO kinetic model of Cu (II) adsorption (Figure 6b) had a very high correlation coefficient value of 0.999, which is better than the PFO kinetic model value of 0.044, indicating that chemisorption is the suggested mechanism that occurs when Cu (II) is adsorbed on the Ag NPs@Bipy-PAN surface [65].

#### 3.4.5. Regeneration and Reusability Effect

**Figure 7:**
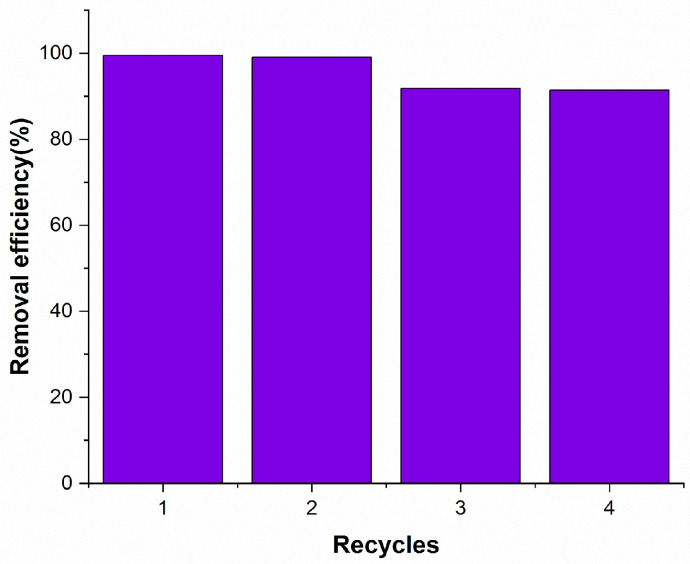
The reusability of Ag NPs@Bipy-PAN (30 mg) on the removal efficiency of 20 mg/L Cu (II) at pH = 7.

The economic aspects of regenerating the adsorbent and its ability to be reused for metal adsorption have great importance. Figure 7 shows the recycling process of Cu (II) adsorption on Ag NPs@Bipy-PAN. It was found that the regeneration process was repeated four times consecutively and the removal efficiency was 99.5% in the first cycle and decreased to 99.1% in the second cycle. Although there was a slight decrease in recycling efficiency during the third cycle, this eventually stabilized. These findings emphasize the significant potential of Ag NPs@Bipy-PAN as an economically viable adsorbent for removing Cu (II) ions, demonstrating remarkable regeneration performance. Consequently, it is highly recommended for water treatment applications. Although a modest decline in performance was observed over four cycles, the chelating Ag–bipyridine interface is expected to limit Ag loss; future optimization—via higher ligand density/crosslinking, pH control, and optional protective interlayers—will be pursued to further suppress leaching and distinguish fouling from true Ag loss.

Overall, the combination of ultrafine particle size, structural stability, and ligand coordination suggests that Ag NPs incorporated into Bipy-PAN could serve as a promising platform for catalytic and functional applications, warranting further studies on stability and reusability [72,73]. Recent research has investigated the application of Ag-based nanomaterials for separation and purification applications, highlighting their efficiency and versatility [74]. The observed ultrafine size and uniform dispersion of Ag NPs within the Bipy-PAN matrix can be rationalized based on the strong coordination interactions between the bipyridine ligands and silver ions. By connecting the material’s structure to its potential applications, these findings provide a more comprehensive understanding of the underlying science.

## 4. Conclusions

In summary, this study reports the successful deployment of Ag NPs@Bipy-PAN as a novel adsorbent for enhancing CO_2_ uptake and cation removal. Bipy-PAN consists of triazine and bipyridine groups connected by aminal (-HN-C-NH-) linkages. The integration of Ag NPs into Bipy-PAN was achieved using the reverse double-solvent method. This unique structure of Bipy-PAN provided abundant active nitrogen sites and a large π-conjugated framework, effectively trapping the Ag NPs within the Bipy-PAN cavities and preventing their aggregation and leaching. The resulting material exhibited an ultrafine average particle size of Ag NPs, measuring 9.2 nm. Furthermore, the inclusion of Ag NPs significantly increased the specific surface area from approximately 161 m^2^/g to 279 m^2^/g, as determined by BET analysis. Remarkably, the Ag NPs@Bipy-PAN material demonstrated exceptional adsorption capacity for CO_2_, outperforming pure Bipy-PAN, with a capacity of 2.99 mmol/g. This improvement can be attributed to the synergistic effect between Ag NPs and CO_2_-philic groups present in the material. Additionally, Ag NPs@Bipy-PAN exhibited a higher adsorption capacity for cations compared to Bipy-PAN. This can be attributed to the enhanced electrostatic interactions between silver metal ions and the increased nitrogen content provided by the additional triazine and bipyridine rings. These modifications resulted in an enlarged number of coordination sites and a higher specific surface area, leading to an enhanced capacity for cationic species. Furthermore, the material demonstrated significant sensitivity in adsorbing Cu (II) ions, achieving a remarkable removal efficiency up to 99% within just 20 min at pH 7 and a concentration of 20 mg/L. This research opens up promising possibilities for utilizing melamine-based porous polyaminals as efficient support materials for immobilizing metal nanoparticles. These materials hold great potential in applications such as CO_2_ adsorption and the removal of heavy metals, contributing to efforts in environmental pollution decontamination.

## Figures and Tables

**Table 1:** The S_BET_ surface area and the CO_2_ uptake of Ag NPs@Bipy-PAN and various melamine-based porous polyaminals.

	S_BET_ (m^2^/g)	CO_2_ Uptake (mmol/g)	Reference
		273 K 1bar	
MPA-1	30	1.74	[63]
MPA-2	33.5	0.98	[63]
MBMOP-1	747	2.56	[64]
MBMOP-2	1082	2.81	[64]
Bipy-PAN	161	1.02	[58]
Ag NPs@Bipy-PAN	279	2.99	This work

S_BET_: Brunauer–Emmett–Teller specific surface area.

**Table 2:** Adsorption capacity and removal efficiency of Bipy-PAN and Ag NPs@Bipy-PAN for several metal ions at pH = 6, with 20 mg of adsorbent and a 20 ppm ion concentration.

	Bipy-PAN		Ag NPs@Bipy-PAN	
Metal Ion	*q_e_* (mg/g)	R%	*q_e_* (mg/g)	R%
Cu (II)	20.52	20.5	93.415	93.6
Pb (II)	68.7	41.16	88.66	88.405
Cd (II)	6.3	6.256	73.06	71.07
Ni (II)	6.2	6.114	68.19	66.624
Ba (II)	7.45	7.45	44	42.389

*q_e_*: adsorption capacity; R%: removal efficiency.

**Table 3:** Comparison of the adsorption capacity of Cu (II) by Ag NPs@Bipy-PAN with the various types of melamine-based porous polyaminal adsorbents.

Adsorbent	pH	Dose (mg)	S_BET_ (m^2^/g)	*q_e_* (mg/g)	Reference
SNW	3.5	150	548	72.9	[66]
MA-Py	5	50	424	5.44	[43]
MA-iPh	5	50	192	1.20	[43]
MA-Th	5	50	34	6.22	[43]
Bipy-PAN	6	30	161	20.52	This work
Ag NPs@Bipy-PAN	7	30	279	98.20	This work

S_BET_: Brunauer–Emmett–Teller specific surface area; *q_e_*_:_ adsorption capacity.

**Table 4:** The adsorption parameters of Cu (II) by Ag NPs@Bipy-PAN based on the Langmuir and Freundlich isotherm models.

	Langmuir Model			Freundlich Model		
Adsorbent	$q_{m}\;(mg/g)$	B	$R^{2}$	*N*	$k_{f}$	$R^{2}$
Ag NPs@Bipy-PAN	13.700	4.918	0.208	3.042	0.453	0.936

**Table 5:** Fitting results of Cu (II) adsorption by Ag NPs@Bipy-PAN to pseudo-first order and pseudo-second-order kinetic models.

	Pseudo-First-Order Model			Pseudo-Second-Order Model		
Adsorbent	*q_e_* (mg/g)	K_1_ $({min}^{-1}$)	$R^{2}$	*q_e_* (mg/g)	K_2_ (g/mg min)	$R^{2}$
Ag NPs@Bipy-PAN	1.224	$-1\times 10^{-5}$	0.044	92.421	0.044	0.999

## Data Availability

The original contributions presented in the study are included in the article, further inquiries can be directed to the corresponding author.
